# Convergent and divergent intra- and internetwork connectivity in Parkinson’s disease with wearing-off

**DOI:** 10.1007/s10072-023-07005-2

**Published:** 2023-08-14

**Authors:** Heng Zhai, Wenliang Fan, Yan Xiao, Zhipeng Zhu, Ying Ding, Chentao He, Wei Zhang, Yan Xu, Yuhu Zhang

**Affiliations:** 1 Department of Neurology, Guangdong Neuroscience Institute, Guangdong Provincial People’s Hospital (Guangdong Academy of Medical Sciences), Southern Medical University, Guangdong Province, Guangzhou, 510080 China; 2 Department of Neurology, Union Hospital, Tongji Medical College, Huazhong University of Science and Technology, Wuhan, 430022 Hubei Province China; 3 Department of Radiology, Union hospital, Tongji Medical College, Huazhong University of Science and Technology, Wuhan, 430022 Hubei Province China; 4 Hubei Province Key Laboratory of Molecular Imaging, Wuhan, 430022 Hubei Province China; 5 Guangzhou Key Laboratory of Diagnosis and Treatment for Neurodegenerative Diseases, Guangdong Provincial People’s Hospital, Guangdong Academy of Medical Sciences, Guangzhou, 510080 China; 6 Guangdong Provincial Key Laboratory of Artificial Intelligence in Medical Image Analysis and Application, Guangdong Provincial People’s Hospital, Guangdong Academy of Medical Sciences, Guangzhou, 510080 China

**Keywords:** Wearing-off, Parkinson’s disease, Functional connectivity, Intranetwork, Internet work

## Abstract

**Objective:**

Our study aimed to explore the functional connectivity alterations between cortical nodes of resting-state networks in Parkinson’s disease (PD) patients with wearing-off (WO) at different levels.

**Methods:**

Resting-state functional magnetic resonance imaging was performed on 36 PD patients without wearing-off (PD-nWO), 30 PD patients with wearing-off (PD-WO), and 35 healthy controls (HCs) to extract functional networks. Integrity, network, and edge levels were calculated for comparison between groups. UPDRS-III, MMSE, MOCA, HAMA, and HAMD scores were collected for further regression analysis.

**Results:**

We observed significantly reduced connectivity strength in the dorsal attention network and limbic network in the PD-WO group compared with the HC group. The PD-WO group showed a decreased degree of functional connectivity at 12 nodes, including the bilateral orbital part of the superior frontal gyrus, right olfactory cortex, left medial orbital part of the superior frontal gyrus, bilateral gyrus rectus, right parahippocampal gyrus, right thalamus, left Heschl’s gyrus, right superior temporal gyrus part of the temporal pole, left middle temporal gyrus part of the temporal pole, and right inferior temporal gyrus. Furthermore, the PD-WO group showed a significantly lower degree of functional connectivity in the left orbital part of the superior frontal gyrus and right gyrus rectus than the PD-nWO group. Internetwork analysis indicated reduced functional connectivity in five pairs of resting-state networks.

**Conclusion:**

Our results demonstrated altered intra- and internetwork connections in PD patients with WO. These findings will facilitate a better understanding of the distinction between the network changes in PD pathophysiology.

## Introduction

Motor fluctuation is considered one of the most important problems in the long-term management of Parkinson’s disease (PD). The initial and common motor complication is wearing-off (WO), which is defined as a predictable recurrence of motor and nonmotor symptoms at the end of a dose of antiparkinsonian medication that usually improves after the next dose [1]. Previous studies have suggested that the degeneration of presynaptic dopamine neurons due to PD progression and fluctuating levodopa levels is likely to give rise to WO [2, 3].

Several functional imaging techniques were used to estimate functional connectivity and nigrostriatal dopaminergic dysfunction in PD patients with motor complications. Previous studies have found that the occurrence of levodopa-induced dyskinesia (LID) is related to anomalous functional networks in the inferior frontal cortex (IFC), basal ganglia (BG), supplementary motor area (SMA), and pre-SMA [4–6]. Other studies used ^18^F-fluorodopa positron emission tomography (PET) have reported that PD patients with WO showed greater dopamine neuronal loss than those without WO [2, 7, 8]. Unlike PET, functional magnetic resonance imaging (fMRI) is a noninvasive technique that can be used to detect aberrant brain functional connectivity and is more convenient for monitoring dynamic changes in the progression of PD.

To our knowledge, there have been no fMRI studies using the blood oxygen level–dependent (BOLD) method to investigate abnormalities in whole-brain functional connectivity in PD patients with wearing-off. Thus, in this study, we aimed to use resting-state fMRI to investigate the changes in whole-brain connectivity at the integrity, network, and edge levels and to assess the relationship between these changes in the patterns of networks in PD patients with wearing-off. We hypothesized that brain functional connectivity would show reduced changes in PD patients with wearing-off. In addition, it is hoped that this study will help us better understand the pathophysiological mechanism and explore potential biomarkers for PD patients with wearing-off.

## Methods

### Patients

This study was approved by the Ethics Committee of Union Hospital, Tongji Medical College of Huazhong University of Science and Technology and Guangdong Provincial People’s Hospital. Informed consent was obtained from all subjects. The study was also conducted in accordance with the Declaration of Helsinki. Sixty-eight patients with PD were recruited between September 2020 and May 2022. Thirty-five healthy controls (HCs) matched by age, gender, and education level were selected. The inclusion criteria were as follows: (1) patients diagnosed with PD according to the 2015 Movement Disorder Society clinical diagnostic criteria [9]; *(2) right-handed Chinese natives; (3) received levodopa and/or dopamine agonist (DA) therapy for 6 months or longer; (4) patients without dyskinesias; and (5) patients whose Parkinsonism was induced by cerebrovascular disease, medications, encephalitis, poisoning, trauma, and other neurodegenerative diseases were excluded. HCs were included without any neurological conditions such as stroke, brain tumor, severe mental disorder, and white matter lesions more than 1 mm visible on structural MRI.

Baseline demographic and clinical data including age, gender, education level, disease duration, Hoehn and Yahr scale (H&Y), unified Parkinson’s disease rating scale (UPDRS) [10] score, and use of anti-Parkinson medication were recorded. Neurological examinations of cognitive and affective disorder states were evaluated by the mini-mental state examination (MMSE) [11], Montreal cognitive assessment (MoCA) [12], Hamilton rating scale for depression (HAMD) [13], and Hamilton rating scale for anxiety (HAMA) [14]. For PD patients, the third part of the UPDRS (UPDRS-III) was used to assess the severity of motor symptoms and evaluated in the daytime during their ON (approximately 1 h after the dose of medication) and OFF (approximately 12 h after the dose of medication) phases [15]. All PD patients were receiving stable dopaminergic therapy prior to the assessment. The levodopa equivalent daily dose (LEDD) was calculated for each PD patient [16]. The validated Chinese version of the 9-item wearing-off questionnaire (WOQ-9) was applied for screening wearing-off, and at least one improved symptom after next dose of medication indicated a diagnosis of WO [17]. The daily off time of the WO group was also assessed using UPDRS item 4.3. Then, PD patients were classified into two groups: patients without wearing-off (PD-nWO) and patients with wearing-off (PD-WO).

### Magnetic resonance imaging acquisition

All patients were performed on a 3.0 Tesla MRI scanner (MAGNETOM Skyra; Siemens Healthcare, Erlangen, Germany) using a 20-channel head/neck coil. All participants were required to lie still in the supine position. Ear plugs were used to reduce the loud noise generated by the scanner, and tight foam padding was used to minimize head motion. Dopaminergic medication was withdrawn overnight for 12 h prior to the MRI scan. Structural three-dimensional T1-weighted images (3D-T1WI) were obtained using a volumetric 3D magnetization–prepared rapid gradient-echo (MP-RAGE) sequence with following parameters: repetition time (TR)=2400 ms, echo time (TE)=2.26 ms, flip angle (FA) =8°, field of view (FOV) = 256×256 mm^2^, voxel size=1×1×1 mm^3^, slice thickness=1 mm, slice number=192, and matrix size=256×256. Resting-state MRI was acquired using a T2*-weighted gradient-recalled echo-planar imaging (EPI) sequence with the following parameters: TR=2000 ms, TE=30 ms, FOV=234×224 mm^2^, slice thickness=3.5 mm, voxel size=3.5×3.5×3.5 mm, and slice number=35. Each functional run contained 240 image volumes for each participant.

### Magnetic resonance imaging data processing

Preprocessing of the fMRI data was carried out using the data processing and analysis for brain imaging (DPABI [18]) software toolbox based on SPM12 [18]. The first ten volumes of each functional section were removed due to signal equilibrium. The remaining 230 volumes were then preprocessed, including slice timing correction using the middle slice as a reference, head motion correction, spatial normalization into the standard Montreal Neurological Institute (MNI) template, resampling to 3 mm × 3 mm × 3 mm voxel size, and spatial smoothing using a Gaussian kernel of full-width at half-maximum (FWHM) 4 mm. Linear regression with nuisance covariates including six head motion parameters, cerebrospinal fluid (CSF) signal and white matter (WM) signal were removed. Moreover, bandpass filtering with a frequency of 0.01–0.08 Hz was applied to reduce the effect of frequency drift. To further minimize the effects of head motion, participants with a mean framewise displacement (FD) > 0.5 mm or cumulative translation of more than 3 mm or rotation of more than 3° were excluded [18].

### Functional connectivity analyses

To analyze the functional connectivity patterns of brain networks, the whole brain (excluding the cerebellum) was divided into 90 regions of interest (ROIs) based on the automated anatomical labeling (AAL) atlas [19]. According to a previous study [20], the 90 AAL regions were clustered into 7 networks: the visual network (VSN), somatomotor network (SMN), dorsal attention network (DAN), ventral attention network (VAN), limbic network (LBN), frontoparietal network (FPN), and default mode network (DMN). Moreover, we also clustered the eighth network named the deep gray matter network (DGN) [21]. The core brain regions constituting the DGN were the bilateral caudate, putamen, pallidum, and thalamus [22–24]. In each of the 90 ROIs, the mean time of each brain region was obtained by averaging the times of all voxels within each of the AAL region to represent the activity of the brain region; i.e., 90 (ROI number) × 230 (time points) were obtained for each subject [25]. The correlation between each pair of regions was measured by calculating the Pearson’s correlation coefficient (*r* values) of the mean time series (size: 90 × 90) for the three groups of patients. Then, the correlation coefficients were converted to the nodal connectivity degree *η* values using an exponential method related to the connectivity “distance” between the two connected ROIs (node i and node j), i.e., *η*_ij_=${e}^{-\xi {d}_{\textrm{ij}}}$, where *ξ*=2 according to previous studies [26, 27]. This method measures how the functional strength of the relationship decreases with the distance between the two nodes (in this case, the network node is the brain ROI), and *d*_ij_= (1-*r*_ij_)/ (1+ *r*_ij_) represents the distance between two seed regions, where *r*_ij_ is Pearson’s correlation coefficient [22, 25].

To investigate the shared general and distinct specific connectivity patterns in the HC, PD with and without WO groups, we studied intranetwork and internetwork connections in the subjects at three levels, including the integrity level, network level and edge level, while the network level included intra- and internetwork analysis [22, 23, 28]. As previously described, the integrity level represents the information flow from the whole brain in a specific node, and this measure is equivalent to the “degree centrality” in graph theory. The total connectivity degree of node i was calculated using *Γ*_*i*_ = ${\sum}_{j=1}^n{\eta}_{\textrm{ij}}$, where *Γ*_*i*_ indicates the sum of all connectivity degrees between *i* and all other nodes of the brain [22, 27]. Intranetwork analysis measures functional connectivity by averaging the transformed correlation coefficients of all ROI pairs within a network, while internetwork analysis measures the functional connectivity of all ROIs between 2 networks. Briefly, the intranetwork measure was calculated using *c*^*n*^= 〈*η*_ij_〉, where *i* and *j* refer to an ROI pair within network *N* and 〈〉 represents the mean across the examined ROI pairs. The internetwork measure was calculated using *c*^*X*, *Y*^= 〈*η*_ij_〉, where *X* and *Y* represent the subnetworks of the eight RSNs, i refers to an ROI in *X*, *j* refers to an ROI in *Y*, and 〈〉 represents the mean across the examined ROI pairs. At the edge level, the functional connectivity was investigated between all the possible connections for each subnetwork pair. Therefore, we explored the pattern of connectivity between 4005 (90×89/2) pairs of ROIs [22, 25].

### Statistical analysis

Statistical analysis was performed using Statistic Package for Social Science (SPSS) software version 23.0. Continuous variables are presented as the means and standard deviations. Categorical variables were expressed as counts and percentages. Independent samples *t* test or Mann–Whitney *U* test was applied to compare two groups. The chi-squared test was used to compare categorical variables. The network-based statistics (NBS) approach (through permutation tests with 10,000 iterations) was used to detect altered connectivity in the whole-brain functional network between the three groups. One-way analysis of variance (ANOVA) was used to compare clinical characteristics and the *Z* scores at three levels among the HC, PD-nWO, and PD-WO groups after controlling for age, gender and education level by using a general linear model (*P* < 0.05). A post hoc Student’s *t* test was then performed for each pair of groups to further investigate the differences and to determine the presence of any statistical significance in ANOVA (*P* < 0.05). The statistical significance of the observed effects was corrected using false discovery rate (FDR) correction. Furthermore, we classified the WOQ-9 score into motor and nonmotor wearing-off scores. Spearman correlation analyses were performed to assess relationships between the altered intra- or internetwork connectivity and clinical variables (duration, LEDD, WOQ-9 score, UPDRS-III, MMSE, MoCA, HAMA, and HAMD scores) of PD patients with wearing-off with age, gender and education level as covariates [23, 29]. A *P*-value less than 0.05 was considered statistically significant.

## Results

### Characteristics of the patients

A total of 36 PD-nWO patients, 30 PD-WO patients, and 35 healthy controls were eventually included in the analysis. The demographic and clinical characteristics of the three groups are listed in Table 1. No significant differences were observed in age, gender, or education level between the HC, PD-nWO, and PD-WO groups (*P* > 0.05). The mean age of the PD-WO, PD-nWO and HC groups were 67.47±9.10, 68.22 ± 8.00, and 66.83±8.11 (mean ± SD) years, respectively (*P* = 0.782). In the PD patients with WO, the mean onset age was younger (62.10±9.81 versus 66.94±7.85 years), with a longer disease duration (5.35±2.89 versus 1.88±1.17 years) and higher *H*&*Y* scores (2.93±0.99 versus 1.54±0.53 years) than those without WO (all *P* < 0.05). In terms of cognitive performance, patients with WO had poorer scores measured with the MMSE and MoCA scores than patients without WO or HC subjects (*P* < 0.01). The scores of HAMA and HAMD in PD patients with and without WO were significantly higher than those in healthy controls (*P* < 0.01). In addition, patients with WO had higher total LEDD (526.43 ± 367.73 versus 310.26±121.55 mg) and UPDRS-III scores (OFF: 38.27±14.33 versus 24.42±12.70, ON: 22.47±10.28 versus 14.22±10.58) than those without WO (*P* < 0.01). In the WO group, the mean daily off time was 5.18 ± 2.02 h.

**Table 1:** Clinical and demographic characteristics of HC, PD-nWO and PD-WO

	HC	PD-nWO	PD-WO	*P*-value
	*n*=35	*n*=36	*n*=30	
Age (years)	66.83 ± 8.11	68.22 ± 8.00	67.47 ± 9.10	0.782^a^
Gender (male/female)	13/22	18/18	13/17	0.550^b^
Education level (years)	7.77 ± 4.33	8.08 ± 4.21	8.33 ± 4.13	0.866^a^
Body weight (kg)	62.83 ± 9.39	62.74 ± 8.94	62.40 ± 9.96	0.981^a^
Age at symptom onset (years)	N/A	66.94 ± 7.85	62.10 ± 9.81	0.031^c*^
Disease duration (years)	N/A	1.88 ± 1.17	5.35 ± 2.89	0.000^d*^
Hoehn & Yahr stage	N/A	1.54 ± 0.53	2.93 ± 0.99	0.000^d*^
UPDRS part I score	N/A	7.08 ± 4.75	7.34 ± 4.58	0.834^d^
UPDRS part II score	N/A	8.33 ± 6.18	14.93 ± 8.97	0.000^c*^
UPDRS part IIII score (OFF phase)	N/A	24.42 ± 12.70	38.27 ± 14.33	0.000^c*^
UPDRS part IIII score (ON phase)	N/A	14.22 ± 10.58	22.47 ± 10.28	0.000^d*^
WOQ-9 motor score	N/A	N/A	3.00 ± 1.11	N/A
WOQ-9 nonmotor score	N/A	N/A	1.03 ± 1.89	N/A
MMSE scores	26.31 ± 2.03	24.94 ± 3.17^1^	22.33 ± 6.19^23^	0.000^a*^
MoCA scores	21.09 ± 3.19	18.92 ± 5.54^1^	16.43 ± 6.46^2^	0.002^a*^
HAMA scores	5.11 ± 5.26	10.31 ± 8.95^1^	10.83 ± 8.48^2^	0.004^a*^
HAMD scores	8.09 ± 9.16	13.11 ± 9.98^1^	15.30 ± 7.64^2^	0.005^a*^
LEDD (mg per day)	N/A	310.26 ± 121.55	594.85 ± 336.86	0.000^d*^
Medication				
Levodopa, *n*	N/A	31	29	0.261^b^
Dopamine agonists, *n*	N/A	30	25	1.000^b^
COMT inhibitors, *n*	N/A	4	9	0.055^b^
MAO B inhibitors, *n*	N/A	2	2	1.000^b^
Amantadine, *n*	N/A	0	1	0.455^b^
Daily OFF time (h)	N/A	N/A	5.18 ± 2.02	N/A

Data are expressed as mean ± standard deviation or number

*HC* healthy controls, *PD* Parkinson’s disease, *WO* wearing-off, *UPDRS* unified Parkinson’s disease rating scale, *WOQ-9* wearing-off questionnaire 9 items, *MMSE* mini-mental state examination, *MoCA* Montreal cognitive assessment, *HAMA* Hamilton anxiety scale, *HAMD* Hamilton depression scale, *LEDD* levodopa equivalent daily dose

^*^P < 0.05 was considered significant differences

^a^*P* values are from One-way ANOVA. Post hoc comparisons further revealed the source of ANOVA derived differences (1: PD non-WO vs. HC; 2: PD-WO vs. HC; 3: PD non-WO vs. PD-WO)

^b^*P* values are from Chi-squared test

^c^*P* values are from two-sample *t* test

^d^*P* values are from Mann–Whitney *U* test

### Integrity level analysis

**Fig. 1:**
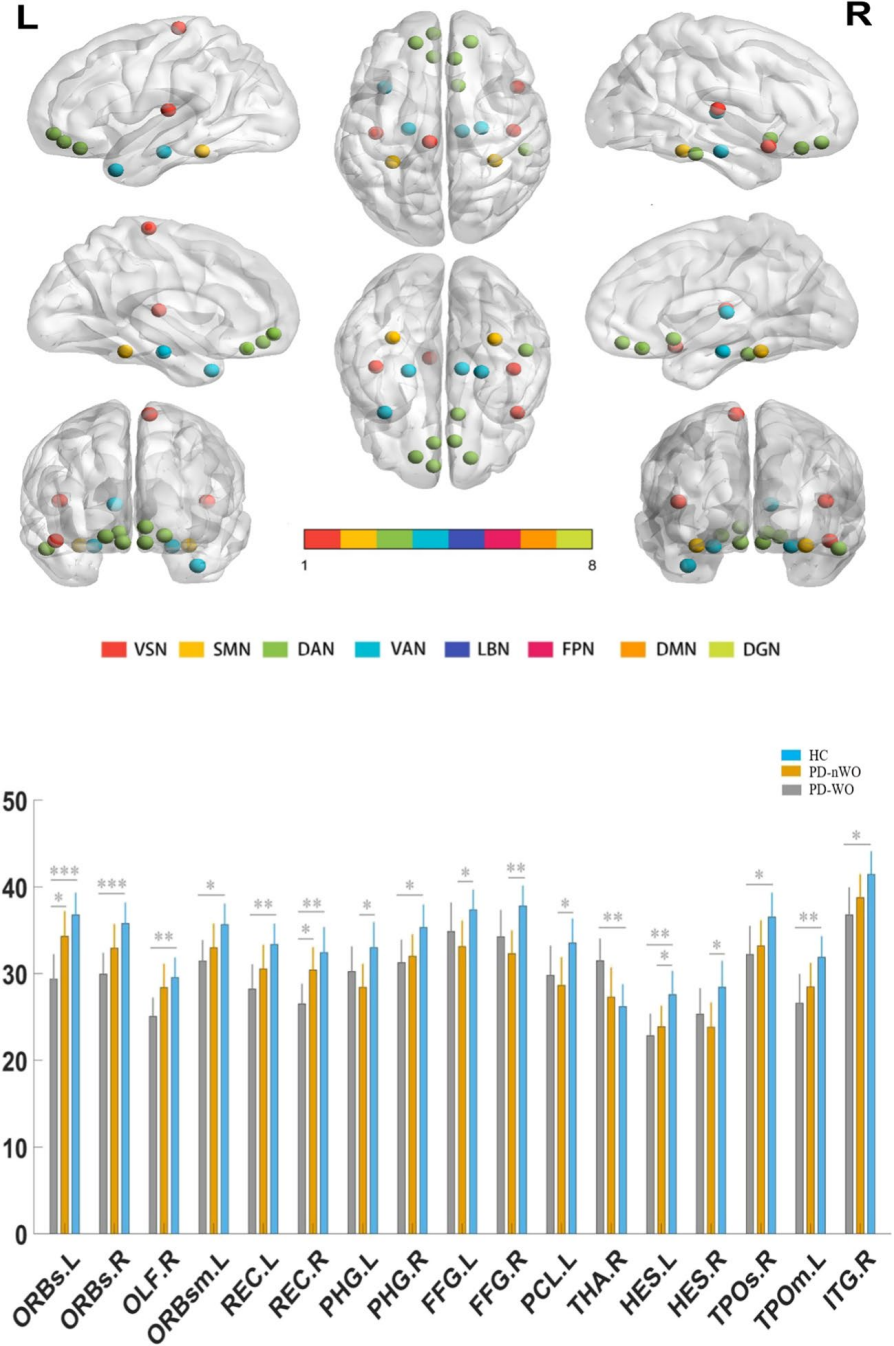
Results for analysis of variance and post hoc comparisons the total degree of connectivity between nodes at the integrity level among the HC, PD non-WO and PD-WO groups. Regions of interest (ROIs) that were statistically significant (*P* < 0.05) in ANOVA are displayed on the brain surface. ROIs from the same RSNs are represented as the same color. Statistical analysis indicates significant differences regarding the total functional connectivity at the 17 nodes distributed in the VSN, SMN, DAN, and LBN (*P* < 0.05, FDR corrected). Histogram showing comparisons of the mean values of the total degree of connectivity of the nodes among three groups (*P < 0.05, **P < 0.01, ***P < 0.001). Abbreviations: VSN, the visual network; SMN, somatomotor network; DAN, dorsal attention network; VAN, ventral attention network; LBN, limbic network; FPN, frontoparietal network; DMN, default mode network; DGN, deep gray matter network; FDR, false discovery rate; ROI, regions of interest; RSN, resting-state network; ORBs, orbital part of superior frontal gyrus; OLF, olfactory cortex; ORBsm, medial orbital part of superior frontal gyrus; REC, gyrus rectus; PHG, parahippocampal gyrus; FFG, fusiform gyrus; PCL, paracentral lobule; THA, thalamus; HES, Heschl’s gyrus; TPOs, superior temporal gyrus part of temporal pole; TPOm, middle temporal gyrus part of temporal pole; ITG, inferior temporal gyrus; L, left hemisphere; R, right hemisphere

At the integrity level, the analysis results showed significant differences in the degree of total functional connectivity at the 17 nodes distributed within the VSN, SMN, DAN, and LBN between the HC, PD-nWO, and PD-WO groups (*P* < 0.05, false discovery rate (FDR) corrected) (Fig. 1). A histogram was used to show the comparisons between the mean values of the total degree of connectivity of the nodes (Fig. 1). Post hoc comparisons further indicated that the PD-nWO group showed significant alterations in the left parahippocampal gyrus (PHG.L), bilateral fusiform gyrus (FFG), left paracentral lobule (PCL.L), and bilateral Heschl’s gyrus (HES) than the results found in the HC group. Compared to the HC group, the PD-WO group showed significant alterations in the bilateral orbital part of the superior frontal gyrus (ORBs), right olfactory cortex (OLF.R), left medial orbital part of the superior frontal gyrus (ORBsm.L), bilateral gyrus rectus (REC), right parahippocampal gyrus (PHG.R), right thalamus (THA.R), left Heschl’s gyrus (HES.L), right superior temporal gyrus part of the temporal pole (TPOs.R), left-middle temporal gyrus part of the temporal pole (TPOm.L), and right inferior temporal gyrus (ITG.R). Furthermore, the PD-WO group showed a significantly decreased degree of functional connectivity in the left orbital part of the superior frontal gyrus (ORBs.L) and right gyrus rectus (REC.R) than was observed in the PD-nWO group.

### Intranetwork connectivity analysis

**Fig. 2:**
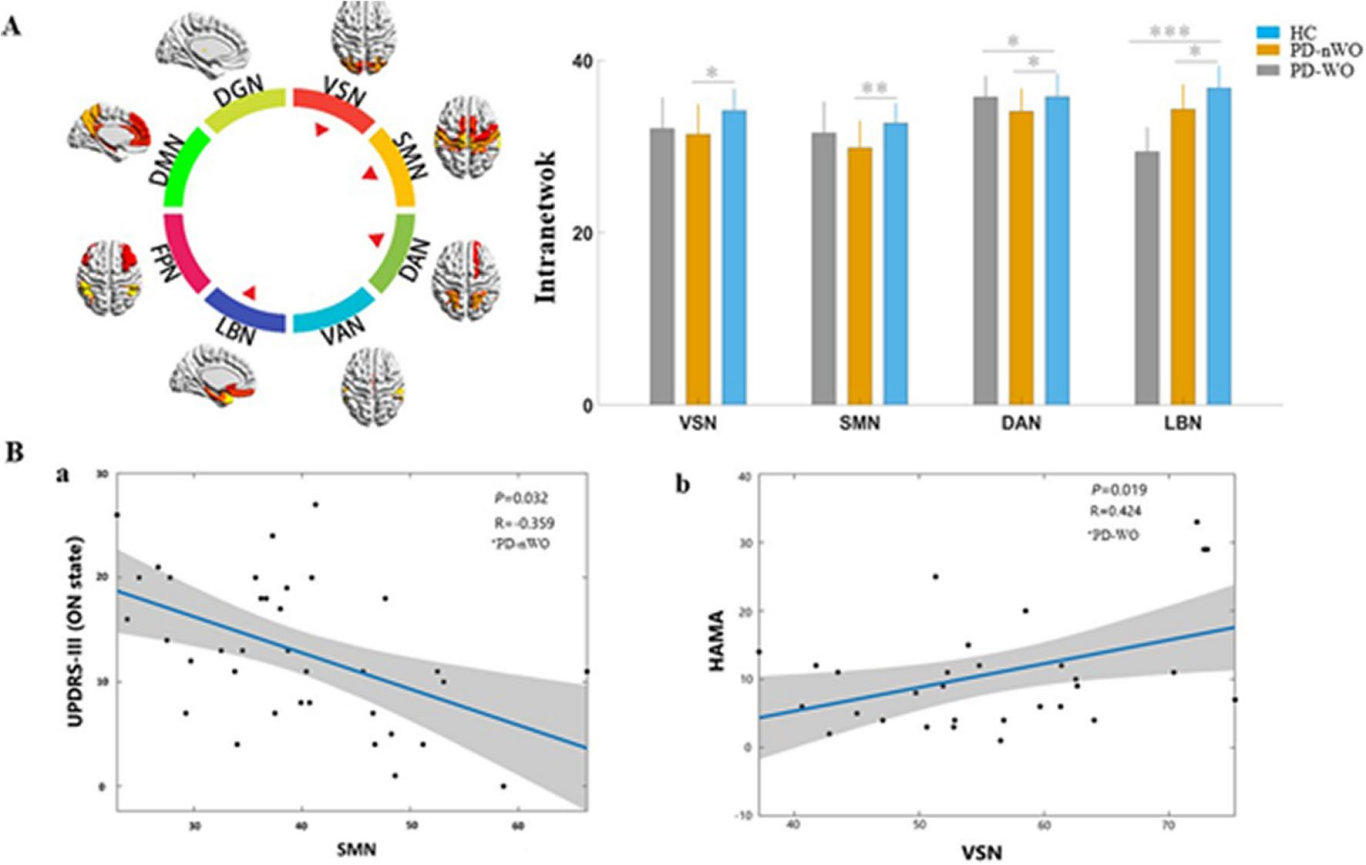
Results for connectivity among the three groups at the intranetwork. **A** The colored circle indicates the composite of 8 RSNs. The red triangles indicate that the RSNs showed altered intranetwork connectivity based on a result showing statistical significance in ANOVA. Histograms showing comparisons of altered intranetwork connections among three groups based on a statistically significant result in ANOVA. The *y*-axis represents intranetwork functional connectivity strength. Asterisks indicate significant group differences (*P < 0.05, **P < 0.01, ***P < 0.001). **B** Scattergrams depicting the relationships between the altered intranetwork connectivity and the clinical performance in PD-nWO and PD-WO groups. Pearson’s correlation coefficients (*R*) and *P* values are reported at the top of each graph. Significant correlations between the UPDRS-III scores (ON state) and intranetwork connection of the SMN in the PD-nWO group (**a**). Significant correlations between the HAMA scores and intranetwork connection of the VSN in PD-WO group (**b**). The *x*-axis represents the intranetwork functional connectivity strength. Abbreviations: RSNs, resting-state network; PD, Parkinson’s disease; WO, wearing-off; VSN, visual network; SMN, somatomotor network; UPDRS-III, motor section of the unified Parkinson’s disease rating scale; HAMA, Hamilton anxiety scale

Statistical analysis showed a significant reduced intranetwork connections in the VSN, SMN, DAN, and LBN in PD patients without WO compared with healthy controls. The PD-WO group showed significantly reduced connectivity in the DAN and LBN compared to the HC group. When comparing the mean connectivity values, ordered reductions (HC > PD-nWO > PD-WO) were observed in intra-LBN connectivity. There were no significant differences in the VAN, FPN, DMN, and DGN between the three groups. Additionally, no significant differences were found in eight intranetwork connections between PD patients with and without WO **(**Fig. 2A**)**.

### Internetwork connectivity analysis

**Fig. 3:**
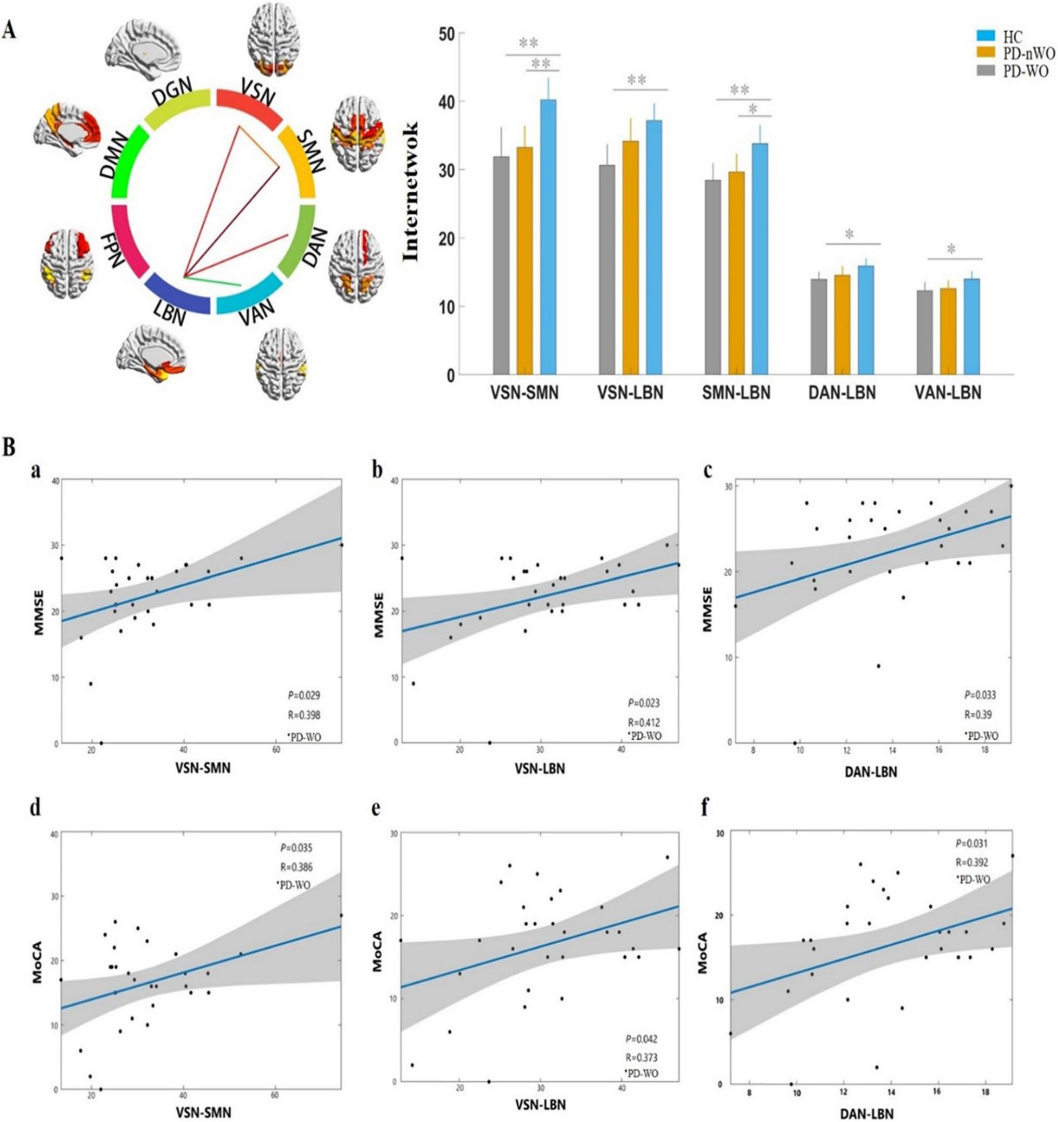
Results for connectivity among the three groups at the internetwork. **A** Color circle showing 8 RSNs. The line indicates altered individual internetwork connectivity based on statistically significant results in ANOVA. Histograms showing comparisons of internetwork connections among three groups. The *y*-axis represents the internetwork functional connectivity strength. Asterisks indicate significant group differences (*P < 0.05, **P < 0.01, ***P < 0.001). **B** Scattergrams depicting the relationships between the altered internetwork connectivity and the clinical performance in PD-WO group. Pearson’s correlation coefficients (*R*) and *P* values are reported at each graph. Significant correlations between the HAMA scores and internetwork connection of the VSN-SMN, VSN-LBN, and DAN-LBN in the PD-WO group (**a, b, c**). Significant correlations between the MoCA scores and internetwork connection of the VSN-SMA, VSN-LBN, and DAN-LBN in PD-WO group (**d**, **e**, **f**). The *x*-axis represents the internetwork functional connectivity strength. Abbreviations: RSNs, resting-state network; PD, Parkinson’s disease; WO, wearing-off; HAMA, Hamilton Anxiety Scale; MoCA, Montreal Cognitive Assessment; VSN, visual network; SMN, somatomotor network; DAN, dorsal attention network; LBN, limbic network

Compared to the HC group, a significant reduction in internetwork connections between the VSN and SMN, and between SMN and LBN were observed in PD patients without WO. The PD-WO group showed significantly reduced internetwork connections between the VSN and SMN, and between the LBN and VSN/SMN/DAN/VAN compared with the results found in the HC group (Fig. 3A**)**. However, no significant differences were found in the eight internetwork connections between PD patients with and without WO.

### Edge level

**Fig. 4:**
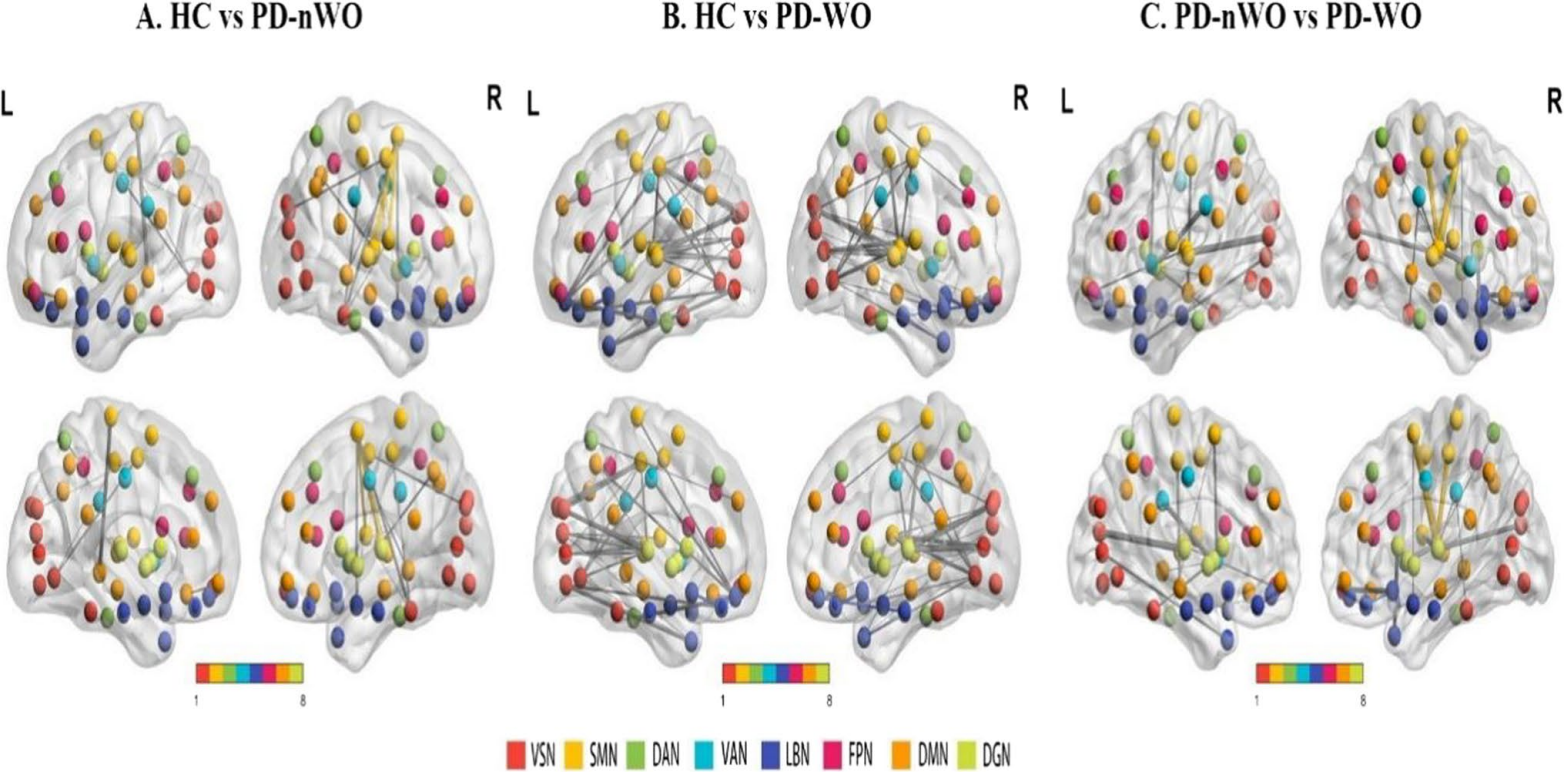
Results for the total degree of connectivity between nodes at the edge level. 388 ROI pairs showed altered connectivity between HC and PD-nWO groups (**A**); 626 ROI pairs showed altered connectivity between HC and PD-WO groups (**B**); 215 ROI pairs showed altered connectivity between PD-nWO and PD-WO groups (**C**) (all *p* < 0.05, FDR corrected). The same color of spheres represented ROIs were from the same RSNs. Abbreviations: ROI, regions of interest; HC, healthy control; PD, Parkinson’s disease; WO, wearing-off; nWO, non-wearing-off; FDR, false discovery rate; L, left hemisphere; R, right hemisphere

Compared with healthy controls, 388 ROI pairs were significantly altered in the PD-nWO group (Fig. 4A); the PD-WO group showed altered functional connectivity in 626 ROI pairs, i.e., 15.63% of the 4005 analyzed ROI pairs (Fig. 4B). Compared with the PD-nWO group, 215 ROI pairs were significantly altered in the PD-WO group (Fig. 4C). These altered pairs were mainly distributed both inside and outside of the VSN, SMN, DAN, VAN, and LBN networks.

### Correlation analysis

**Fig. 5:**
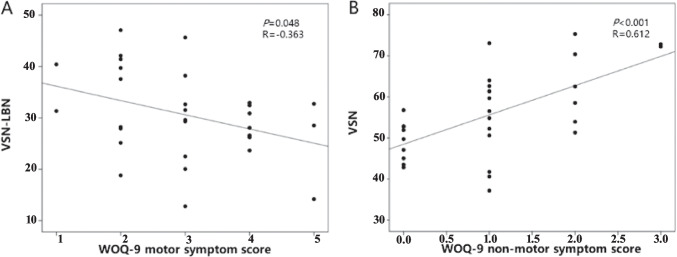
The correlation plots of altered connectivity and WOQ-9 scores. **A** Correlations between the motor wearing-off score and internetwork connections between the VSN and LBN in the PD-WO group. **B** Correlations between the nonmotor wearing-off score and intranetwork connections of the VSN in the PD-WO group. The *y*-axis represents the intra- or internetwork functional connectivity strength. PD, Parkinson’s disease; VSN, visual network; LBN, limbic network; WOQ-9, wearing-off questionnaire 9 items

We performed Pearson correlation analyses between the significantly altered connectivity and clinical information. The results showed that intranetwork connectivity in the SMN was significantly correlated with UPDRS part III scores (ON phase) in the PD patients without WO (*r*= −0.359, *P*= 0.032). Our results also found that intranetwork connectivity in the VSN was significantly correlated with HAMA scores in the PD patients with WO (*r*= 0.424, *P*= 0.019) (Fig. 2B). In addition, significant correlations were found between the HAMA scores and internetwork connections of the VSN-LBN, VSN-SMN, and DAN-LBN, as well as between the MoCA scores and internetwork connections of the VSN-LBN, VSN-SMN, DAN-LBN in the PD patients with WO (Fig. 3B). Significant correlations were noted between the VSN-LBN and motor wearing-off score (*r*=−0.363, *P* = 0.048), and between the VSN and nonmotor wearing-off score (*r*=0.612, *P* < 0.001) (Fig. 5).

**Fig. 6:**
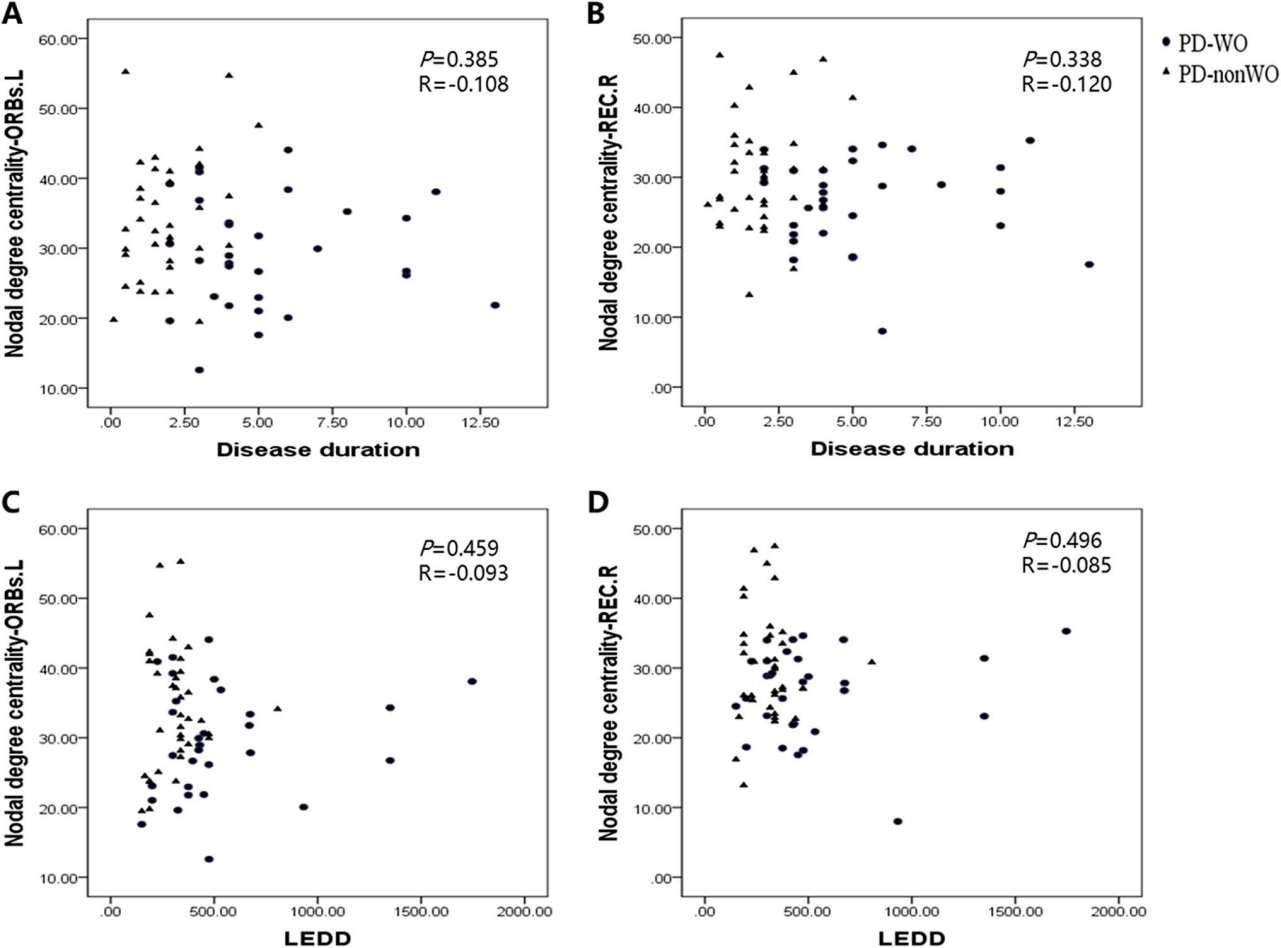
The correlation plots of clinical factors (disease duration, LEDD) and two altered nodal degree centralities in PD-WO group and PD-nWO group. **A, B** Correlations between the disease duration and two altered nodal degree centralities in the PD-WO group and PD-nWO group. **C, D** Correlations between LEDD and two altered nodal degree centralities in the PD-WO group and PD-nWO group. The *y*-axis represents the altered nodal degree centralities strength. PD, Parkinson’s disease; WO, wearing-off; ORBs.L, left orbital part of superior frontal gyrus; REC.R, right gyrus rectus; LEDD, levodopa equivalent daily dose

We also investigated the correlation between other clinical factors (disease duration, LEDD) and two altered nodal degree centralities (ORBs.L and REC.R) in the PD-WO and PD-nWO groups. The vertical axis of Fig. 6 is the nodal degree, and the horizontal axis is the disease duration and LEDD. No correlation was found between duration, LEDD, and two nodal degree centralities in the two groups.

## Discussion

**Fig. 7:**
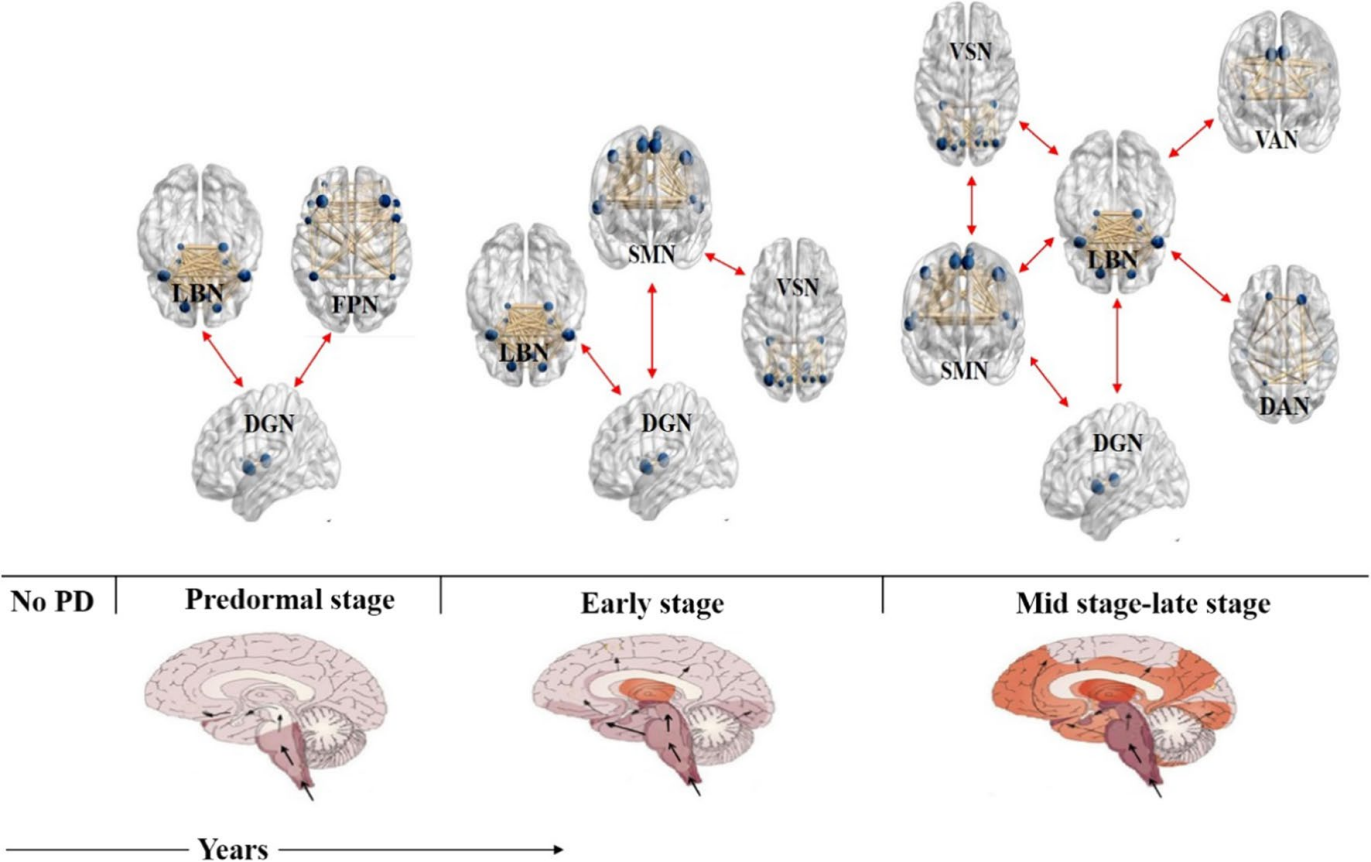
The hypothetical model summarizing our findings how lewy body affects brain networks in PD. Based on the Braak’s hypothesis that Lewy bodies first appear in the brain stem, and spread to the midbrain and the substantia nigra pars compacta, then ascends to involve the cerebral cortex (the darker the shaded area, the earlier it is affected). Wearing-off is the common motor complication and occurred in mid and late stage of PD. We hypothesized that LBN may play a key role in motor and nonmotor symptoms of PD. Red arrows indicating reduced interactions between networks. PD, Parkinson’s disease; WO, wearing-off; VSN, visual network; SMN, somatomotor network; DAN, dorsal attention network; VAN, ventral attention network; LBN, limbic network; FPN, frontoparietal network; DGN, deep gray matter network

In this study, we investigated the changes in resting-state functional connectivity of 90 regions and eight well-defined networks in PD patients with wearing-off. The disruption of the brain networks was evaluated by the intra- and internetwork of resting-state functional connectivity associated with the presence of wearing-off in PD. As the main findings, we observed a decreased degree in the left orbital part of the superior frontal gyrus and right gyrus rectus in PD-WO compared to PD-nWO. Based on the NBS approach, the reduced connections were mainly found in the VSN, SMN, DAN, and LBN between PD-WO patients and healthy controls. A hypothesis model summarizing our findings based on Braak’s hypothesis [30] is shown in Fig. 7. We propose a hypothetical model to explain how Lewy bodies affect brain networks in PD. These findings have promoted our understanding of the underlying neural mechanism divergence from a network perspective during PD progression.

Previous rs-fMRI studies have shown significant alterations in connectivity in PD patients compared with HCs in different cortical and subcortical areas, including the striatum, frontoparietal cortex, gyrus rectus, visual cortex, temporal cortex, and thalamus [31, 32]. Compared with the HC group, the PD-WO group had a significant reduction in connectivity in a set of regions, including the orbital frontal cortex, Heschl’s gyrus, temporal gyrus, and parahippocampal within DAN and LBN. Additionally, internetwork analysis also confirmed that PD-WO patients had abnormal connections between the VSN and SMN, and between the LBN and VSN, SMN, DAN, and VAN. Compared to the PD-nWO group, the PD-WO group demonstrated significantly decreased FC in the left orbital part of the superior frontal gyrus and right gyrus rectus.

Edge-level analysis indicated that widespread connectivity anomalies were found in early PD among the brain networks. In addition, connections between the anterior central gyrus and multiple brain regions of the VSN and LBN were decreased in early PD [23]. Our edge-level analysis showed that extensive intranetwork and internetwork connectivity abnormalities were concentrated in the connections among the VSN, SMN, DAN, VAN, and LBN in PD patients with wearing-off, suggesting that abnormal functional connectivity existed among all stages of PD. These results might help us to better understand the degeneration process of PD.

### Correlations between altered connectivity and clinical information

Demographic factors for WO observed in this study, such as disease duration and LEDD, have been reported to influence the occurrence of wearing-off [3]. Consistent with previous studies, the present results showed that patients with WO have higher levodopa daily dose and longer disease duration. Recent studies have proposed that dopaminergic therapy might impact functional connectivity in PD patients. Danget et al. [33] showed that dopamine drugs enhanced the connectivity between the DMN and the frontoparietal control network in the resting state. Zhang et al. [34] found that dopaminergic medication had a positive effect on the integrity of the DMN in nondemented PD patients in the ON condition. Another study suggested that dopamine drugs might have a specific and dose-dependent influence on both network integrity and task-related activations in PD patients [35]. To further investigate the effect of LEDD and disease duration on the two nodal degrees of the PD-WO group, we used correlation analyses between the LEDD, disease duration, and decrease degree of functional connectivity in the left orbital part of the superior frontal gyrus (ORBs.L) and right gyrus rectus (REC.R). We found no correlation between the strength of functional connectivity in the left ORBs and right REC and the daily LEDD and disease duration in our PD-WO group. Our results must be interpreted with caution since we only used correlation analyses in our PD-WO patients on medication during the off state, and we did not compare on- and off-medication conditions of rs-fMRI changes in each subject.

### Impaired functional connectivity associated with the SMN

Network-based statistic (NBS) analysis demonstrated specific networks of reduced connectivity in PD patients when compared to healthy controls. A previous study showed that FC between the SMN and LBN, and between the VAN and VSN was more prone to damage in all PD patients. The author found that abnormal FC within the VSN correlated with visuospatial dysfunction, and the SMN correlated with the motor severity of disease, which might play a crucial role in the pathogenesis of PD [36]. WO is attributable to the degree of nigrostriatal neurodegeneration and synaptic abnormalities in striatal neurons related to chronic levodopa therapy [2, 3]. With a growing number of studies focused on motor complications in PD, cortico-cortical network alterations have been identified. Studies in structural and rs-fMRI have found that the frontal cortex (including SMA and pre-SMA) was overactive in levodopa-induced dyskinesias (LIDs) patients. Their data suggested that chronic levodopa administration may cause functional abnormalities in specific brain areas [4]. Our data show that the severity of the motor symptoms measured by the UPDRS-III score was related to the functional connectivity of the SMN in the PD-nWO group, indicating that the decreased functional connectivity in the SMN is related to movement disabilities in PD. Although our results are consistent with a previous study, we found that the FC in the SMN was not significantly decreased in the PD-WO group compared with that in the PD-nWO group. We hypothesize that there seems to be aberrant reorganization of neuronal pathways in patients with wearing-off, which is tightly correlated with chronic dopaminergic therapy.

### Impaired functional connectivity associated with the VSN

The VSN is mainly located in the occipital lobe and ventral temporal cortices. Recent evidence suggests that PD patients with visual processing deficits are at higher risk of dementia [37]. Cucca et al. [38] investigated the links between visual impairment, motor deficit and cognitive domains in PD. Impairments in visual perception and processing can significantly impact motor performance. Their results found that a functional reorganization of the visual network was linked to motor improvement in PD. Few studies have shown functional changes inside the right occipitotemporal gyrus [39] and the left inferior occipital gyrus [40] within the VSN in PD patients with freezing of gait. Yu et al. [41] found that the resting-state connectivity between the sensory-motor network and VSN was decreased in late-stage PD. However, the interaction between the VSN and PD-related motor regions in the resting state needs to be further investigated.

### Impaired functional connectivity associated with the LBN

According to previous literature, the LBN has three networks: the hippocampal-diencephalic-retrosplenial network, temporo-amygdala-orbitofrontal network, and default mode network [42]. Previous studies have shown that LBN abnormalities are related to cognitive impairment, anxiety, and depression in PD. Resting-state MRI using regional homogeneity (ReHo) methods showed that abnormal neural activity in the supplementary motor area (SMA) and the prefrontal-limbic network were related to depression in PD [43]. In addition, PD-related changes in the limbic system are linked not only to alterations in emotion processing but also to a more extensive symptom complex including cognitive impairment, sleep disorders, and motor dysfunction. However, the correlation in functional connectivity between the SMN and LBN is less known in PD.

In our study, we also found that the internetwork connections between the VSN and SMN and between the VSN and LBN in the PD-WO group were lower than those observed in the PD-nWO group, although there was no significant difference between the two groups. There was a significant negative correlation between the WOQ-9 motor score and VSN-LBN connectivity, and the nonmotor score was positively correlated with the VSN. We also found a linear relationship between the MMSE/MoCA score and the strength of both VSN-LBN and VSN-SMN connectivity. These findings suggest that functional abnormalities in the LBN and VSN may be involved in the severity of WO symptoms in PD.

### Impaired functional connectivity associated with the VAN and DAN

A recent study demonstrated that dysfunction in fronto-striato-limbic connectivity has been related to freezing of gait [44]. Dynamic connectivity analysis showed that dysfunction of the amygdala was associated with UPDRS scores, indicating that amygdala changes may affect movement through the regulation of affective states [45]. The DAN and VAN are two main attention networks that play a vital role in visuospatial attention and cognitive processing. Visual processing impairment, including misperceptions and hallucinations was closely related to the DAN, VAN, and VSN in late-stage PD [46]. In our study, we found anomalous connections between the LBN and VSN, SMN, and DAN in the PD-WO group, suggesting a possible contribution of subcortical limbic circuit dysfunction in the pathogenesis of cognitive, emotional, and movement symptoms in PD patients who developed wearing-off. These findings may provide evidence of decreased FC between resting-state networks (RSNs) in WO patients. It would be meaningful to explore how other regions interact with the LBN in motor fluctuations in future studies.

## Limitations

There are some limitations that should be considered in the present study. First, the small sample size of our study should be considered when interpreting these findings. However, statistical power may explain FC alterations observed at the whole-brain level of analysis. Therefore, our results are interpretable based on the current sample size. Whether abnormal wearing-off-related network patterns are fixed still requires more research, and a larger sample size and longitudinal follow-up are needed for further investigation. Second, PD patients were medicated with their normal dopaminergic medications during the fMRI experiment, and the disease duration did not match well in our study. Thus, it is not possible to avoid the possible influence of medication and disease duration on the results of this study. Therefore, we have considered this as one of the covariates in the correlation analysis to mitigate its effect. Third, the two PD groups differed across multiple dimensions, including age at onset, disease duration, disease severity levodopa dosing, and cognitive changes. Although we have tried our best to eliminate the influence of these factors in statistical analysis, it may still affect the inference from the difference between the two patient groups or their differences with controls. Finally, functional MRI data were acquired from PD-WO patients during the off state, and further research is needed to detect the potential difference between the on and off states to assess the characteristics of brain networks.

## Conclusions

We used a graph theory approach to detect changes in the brain functional network in PD-WO patients. In conclusion, PD patients with and without wearing-off have different abnormal brain areas. The present study demonstrated that PD-WO patients exhibited a significantly decreased degree of FC in the left orbital part of the superior frontal gyrus and right gyrus rectus compared with those without WO. Moreover, PD patients with wearing-off showed decreased intranetwork connections in the DAN and LBN. The PD-WO group showed significantly reduced internetwork connections between the VSN and SMN, and between the LBN and VSN, SMN, DAN, and VAN. Our study demonstrated that abnormal connectivity between the limbic and other cortical networks was positively associated with the severity of wearing-off symptoms in PD patients, emphasizing that the functional contribution of the LBN may play an important role in the pathophysiological mechanisms of wearing-off in PD.

## Data Availability

Data sharing is allowed for other investigations to replicate the results.
